# Spectral purity, intensity and dominant wavelength: Disparate colour preferences of two Brazilian stingless bee species

**DOI:** 10.1371/journal.pone.0204663

**Published:** 2018-09-28

**Authors:** Sebastian Koethe, Sarah Banysch, Isabel Alves-dos-Santos, Klaus Lunau

**Affiliations:** 1 Institute of Sensory Ecology, Heinrich-Heine-University Düsseldorf, Düsseldorf, Germany; 2 Laboratório de Abelhas, Departamento de Ecologia, Instituto de Biociências, Universidade de São Paulo – USP, São Paulo, São Paulo, Brazil; University of Sussex, UNITED KINGDOM

## Abstract

Bees use floral colour as a major long distance orientation cue. While it is known for bumblebees and honeybees that dominant wavelength (≙ colour hue), colour contrast and spectral purity (≙ saturation) are crucial for flower detection and discrimination, only little is known about colour preferences in stingless bees (Meliponini). In this experiment freely flying workers of two Brazilian species of stingless bees–*Partamona helleri* and *Melipona bicolor*–were tested for colour preferences concerning the colour parameters dominant wavelength, spectral purity and intensity (≙ brightness). Each individual bee had to perform 57 tests, in which a definite series of dual choices between colour stimuli varying in intensity, spectral purity or dominant wavelength were presented. The results show that *P*. *helleri* chose colours of higher spectral purity and preferred bluish colours, while *M*. *bicolor* made generalized colour choices. Intensity did not influence the colour choice of any bee species. The results of *P*. *helleri* are consistent with findings for honeybees and bumblebees, while colour preferences in *M*. *bicolor* seem to be absent.

## Introduction

Stingless bees are considered important pollinators in tropical and subtropical regions. Stingless bees are the most speciose, the most abundant and most diverse group of eusocial bees [1]. The human food consumption worldwide causes the demand for pollination management with native or introduced bees in tropical regions. Introduced bee species like honeybees endanger native species and can lead to extinction of local populations [2–4]. Unlike honeybees only few studies investigate stingless bees and their value for crop pollination [5,6]. Honeybees are the preferred bee species for crop pollination although many stingless bees show comparable characteristics [7]. Stingless bees do not use a dance language like honeybees to share information concerning food sources, but use trophallaxis, excited movements, sound production, body contact, odour traits, chemical markings or visual tracking of nestmates to share information [8,9]. Furthermore, queens of stingless bees are replaced by their offspring leading to a long lifespan for colonies [10,11]. Despite many common features, stingless bees are much more diverse than honeybees (e.g. body size, foraging strategy, and colony size) and are able to provide pollination services that honeybees may not be able to provide (e.g. buzz pollination of flowers with poricidal anthers by *Melipona*, see Sarto et al. [12]). The lack of a functional sting is an additional advantage in particular for enclosed places like green houses or urban areas. Most stingless bees forage in high-density groups on food sources that were located by scout bees [13,14]. High-density foragers can be divided into two groups–non-aggressive foragers, including e.g. *Melipona*, *Partamona* and *Scaptotrigona*, and aggressive foragers like *Trigona* [15]. Aggressive foragers have less scout bees than non-aggressive ones but drive away non-aggressive bees from located food sources [15]. To avoid the loss of a food source, non-aggressive foragers need to exploit their food sources quickly before they are detected by aggressive foragers [15]. The evolutionary pressure to find food sources leads to the question how stingless bees detect flowers. For honeybees and bumblebees many studies confirmed the importance of floral colour for the detection of flowers [16–19]. So far only very few studies analysed colour perception in stingless bees [20–23].

Generally, bees possess three photoreceptor types with maxima at ~340nm ‘UV’, ~430nm ‘blue’ and ~540nm ‘green’ [16, 24,25]. The distribution of photoreceptors can be found among all genera of bees and suggests phylogenetic constraints for bee vision [25,26]. Studies about colour perception in bees have identified important traits of colours that facilitate detection, recognition and discrimination of colours and thus aid colour choice in bees. The main traits of colours that influence bees are dominant wavelength (≙ hue), spectral purity (≙ saturation) and green contrast, while the colour intensity (≙ brightness) is discussed to have no influence on bees’ colour choice [17–19,27–31].

In general, bees appear to have a preference for blue colours, but also preferences for yellow in bumblebees and for UV-absorbing white colours in stingless bees could be observed [17,20,21,28,32]. The chromatic perception of bees depends on the visual angle between bee and target. If the visual angle is below 15° honeybees only perceive colours with their green receptor–also known as green contrast [17]. If the visual angle surpasses 15° honeybees are able to use colour vision [17]. In bumblebees a visual angle of 2.7° is sufficient to perceive colours [19]. The green contrast functions for far-distance detection of flowers, while chromatic contrast functions for close-distance recognition and both are important for flower detection in bees [18,19,33]. The contrast of a colour against the background is an important cue for bees and influences the choice behaviour of bees [17,34]. The size of a target can influence whether bumblebees use green or colour contrast to detect flowers and honeybees’ decisions concerning target shape are influenced by the background colour [18,35]. A study conducted by Spaethe and colleagues [36] found that the discrimination of colours is poorer in *Trigona* cf. *fuscipennis* and *Tetragonula carbonaria* than in honeybees and bumblebees. Furthermore, spectral purity of colours influences the choice of honeybees and bumblebees. When bumblebees and honeybees have to choose between stimuli of the same dominant wavelength but with different values of spectral purity the stimuli with higher spectral purity are preferred over less spectrally pure stimuli [29,30]. These results could not be verified for stingless bees so far [20,21]. Unlike spectral purity, the intensity of colours is assumed to have no influence on bees’ colour choice [31,37,38]. Consequently, colour vision models like the colour hexagon by Chittka [37] take no account of intensity. However, Hempel de Ibarra et al. [39] found, based on experimental data, that an increase of intensity in light stimuli improves colour discrimination of honeybees probably based on contrast between floral colour and background.

In this study, colour choices of two stingless bee species, *Melipona bicolor* and *Partamona helleri* (both belong to the tribe Meliponini) were analysed. The aim is to see whether these two stingless bee species share similar preferences known for honeybees or bumblebees. Do these two stingless bee species prefer specific dominant wavelengths, like honeybees and bumblebees are known to prefer blue colours, and is their choice also depending on spectral purity? Observations in the field showed that many stingless bees forage on red bird-pollinated flowers, although these flowers appear achromatic to bees [40–43]. This might be explained by the use of intensity cues or green contrast for flower recognition in stingless bees. Therefore, we tested freely flying workers of the two stingless bee species following a short training to the test area in a series of dual choice tests in which distinct colour parameters were varied.

## Material and methods

### Production and characteristics of stimuli

**Fig 1:**
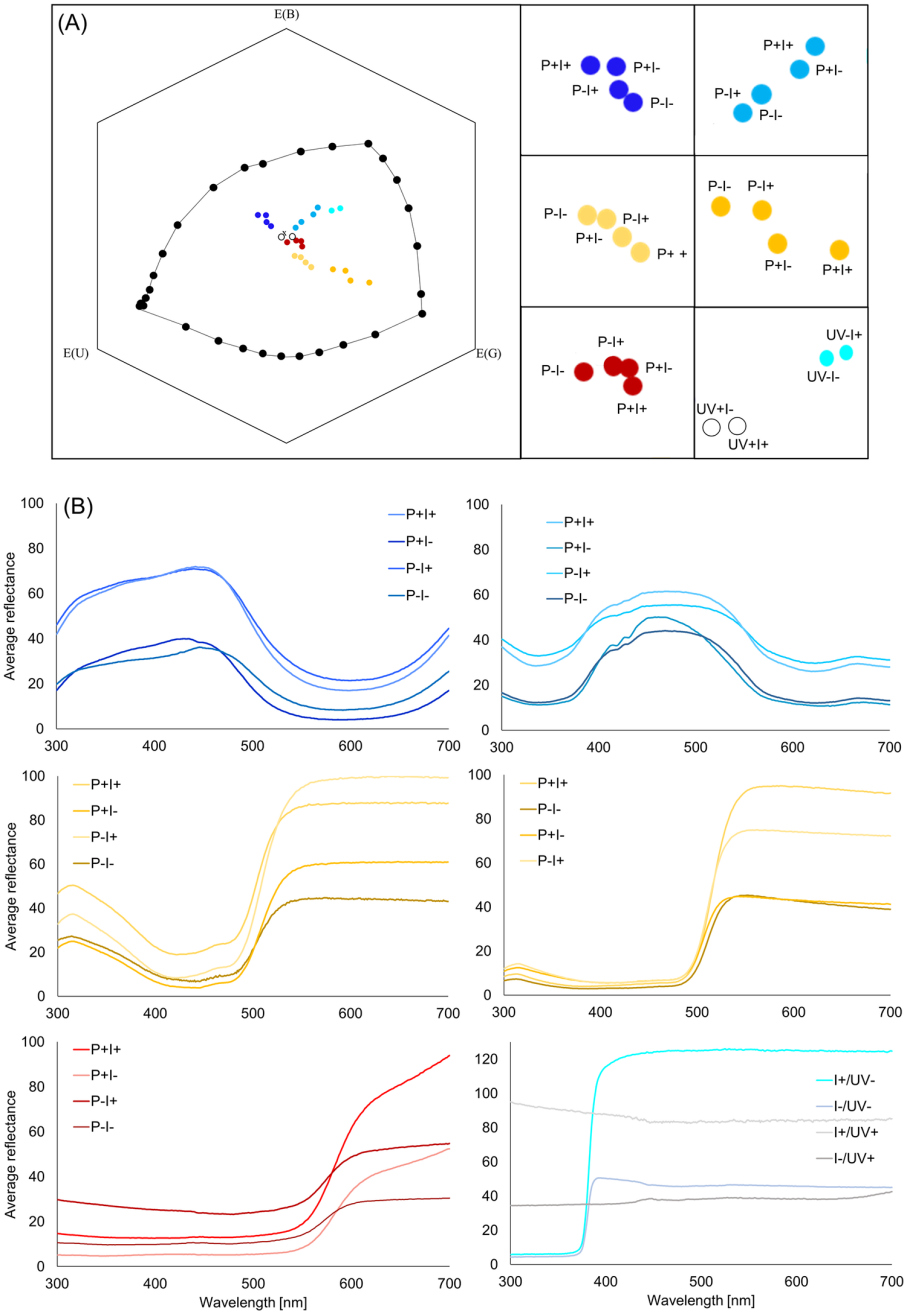
Analysis of used colour stimuli. (A) The colour hexagon according to Chittka [37] displays the perception of colours in accordance with bee-specific photoreceptor sensitivities (*Melipona quadrifasciata*), the background (grey Styrofoam wallpaper) and the ambient light (standard daylight illumination D65) (from top right to bottom left: UV-blue, blue, UV-yellow, yellow, red, white) [16]. (B) Reflectance curves of all colour stimuli that are included in the six colour categories used in the experiments (from top right to bottom left: UV-blue, blue, UV-yellow, yellow, red, white).

The colour stimuli based on a variety of basic colour pigments (Artist Pigments: “Sky Blue”, “Ultramarine Blue”, “Yellow”, “Bright Red Ochre” and “Zinc White”, Art Material International Warenhandelsgesellschaft mbH, Kaltenkirchen, Germany) that were mixed with achromatic pigments (black = “DeiArt Russverkollerung”, Deifel GmbH & Co. KG, Schweinfurt, Germany; white = Barium sulphate, 98% extra pure, Acros Organics BVBA, Geel, Belgium; grey = defined mixture of white and black, see S1 Table). The resulting colours were measured via spectrometer analysis (USB4000 miniature fibre optic spectrometer, Ocean Optics GmbH, Ostfildern, Germany) at an angle of 45° using a UV-NIR deuterium halogen lamp (DH-2000-BAL, Ocean Optics GmbH), which was connected to the spectrometer by a UV–VIS fibre optic cable (Ø 600 μm, QR600-7-UV 125 BX, Ocean Optics GmbH). The obtained spectral data were plotted in the colour hexagon by Chittka [37] (see Fig 1). The receptor-specific contrast (*q*_*i*_) between stimulus and background is calculated based on the quantum flux (*Q*_*i*_) given by:

$$Q_{i}=\int_{300}^{700}S_{i}(\lambda )I(\lambda )D(\lambda )d(\lambda )$$

$$q_{i}=\frac{Q_{i}(stimulus)}{Q_{i}(background)}$$

where *S*_*i*_(λ) refers to the spectral sensitivity function of the photoreceptor type *i* (UV, blue and green) considering the spectral sensitivity of *M*. *quadrifasciata*. *D*(*λ*) is the illumination (here D65 standard illumination) and *d*(λ) denotes the wavelength step size [16].

Based on these results the amount of light absorbed by each photoreceptor type is given by:

$$P=Q_{i}*R$$

where *R* is the sensitivity factor simulating the adaptation of the photoreceptor types to the background (*I*_*B*_):

$$R=1/\int_{300}^{700}S_{i}(\lambda )I_{B}(\lambda )D(\lambda )d(\lambda )$$

The absorption of each photoreceptor (P) can be transduced into photoreceptor excitation (E) by:

E=P/(P+1)

For further analysis of the bees’ choice behaviour, the chromatic contrast was calculated according to the colour hexagon by Chittka [37]. It is defined as the perceptual distance between a colour locus and the background given in hexagon units. The spectral purity results from the perceptual distance between a colour locus and the background in relation to the distance between the background and the spectral line [33].

$$SP=\frac{H_{i}(target-background)}{H_{i}(spectral\;locus-background)}$$

The intensity was calculated by adding up the values of the receptor excitation for all three photoreceptors and dividing those by three [18]. Based on the results of these calculations, four stimuli of each dominant wavelength were selected (S1 Table). In addition to the calculations according to the hexagon, the values for saturation and luminance were calculated according to Valido et al. [44] which are based on the reflectance of stimuli and does not include the photoreceptor sensitivities of the receiver (S1 Table). The pigments were compacted into culture dishes (35 mm diameter, 10 mm height) by using a mechanical press (custom made).

### The experimental setup

For the experimental setup, two PVC panels (50 cm x 50 cm; 50 cm x 25 cm) were connected with a hinge (S1 Fig). The smaller PVC panel was used as a base to stabilise the bigger PVC panel that was fixated at an angle of 45°. A metal plate attached to both PVC panels stabilised the structure. The bigger PVC panel was covered with a grey Styrofoam wallpaper reflecting constantly throughout the UV and visible range of wavelength (Climapor Insulation Wallpaper Graphite Laminated with Pasteboard, Saarpor Klaus Eckhardt GmbH Neunkirchen Kunststoffe KG, Neunkirchen, Germany). Two petri dish lids were affixed to the wallpaper, using Velcro tape, with 5 cm distance to the midpoint of the PVC sheet and functioning as receptacles for the pressed colour stimuli. Below each stimulus a balcony made of metal plate covered with Styrofoam wallpaper was affixed as a landing platform for the bees holding a PCR tube lid in the centre to offer sucrose solution to the bees.

### Bee keeping and conditioning

The hives of *Melipona bicolor* and *Partamona helleri* were located at the campus of Universidade de São Paulo (USP) in the garden of the BeeLab. The nest of *M*. *bicolor* was placed inside the lab with an entrance leading outside while the nest of *P*. *helleri* was located outside of the lab. The workers of both species were freely flying and flower experienced. Gravity feeders with ~10–30% sucrose solution were placed in close proximity to hives of a variety of stingless bees. Most species (*Melipona quadrifasciata*, *Scaptotrigona depilis* and *Trigona spinipes*) were deterred by honeybee workers and only workers of *P*. *helleri* were voluntarily feeding at the feeder. For the training of *P*. *helleri*, workers were caught at the feeder and then trained to forage at the experimental setup. Each worker was labelled with nail polish to identify individuals. Workers of *M*. *bicolor* were trained individually from the entrance of their nest to the experimental setup by leading the way with sucrose solution. Since no recruitment by the bees happened, each worker had to be trained individually and could be tested as such. In total, 24 individuals of *P*. *helleri* and 20 individuals of *M*. *bicolor* were tested.

### The experimental procedure

Prior to the experiment, the bees were trained to visit both balconies of the experimental setup to avoid any effect of the stimuli’s position. During the training no stimuli were offered, only the empty petri dish lids were presented. After a bee had flown several times to both balconies, the experiment started.

A total of 57 definite dual choice tests were offered in a semi-randomised order (see S2 Table) to the bees in which all four stimuli of one colour category were tested against each other (6 tests per colour category, 36 in total) and the seven dominant wavelengths (most intense and spectrally purest stimulus of UV-blue, blue, UV-yellow, yellow, UV-reflecting white, UV-absorbing white and red) were tested against each other (21 tests in total).

The colour categories were mixed in its order and the tests within one colour category were not conducted consecutively. To avoid conditioning caused by the order of tested stimuli, the order was turned around for some of the bees. Each bee made one decision per foraging bout. While the bee returned to the hive the stimuli were changed and the reward refilled.

### Statistical analysis

The statistical program R was used to analyse the data [45]. All data were tested for normal distribution by using the Shapiro-Wilk test.

The pooled data were analysed by testing the bees’ choices for the different stimuli of each colour category using a generalised linear mixed model (GLMM) [46]. We used the “lme4” package of R [47] to analyse the individual choices of the bees, which were assessed using GLMM with binomial distribution of data and the best linear fit depending on akaike information criterion (AIC) score. For the overall test, we analysed the number of choices for each stimulus as fixed effect and each individual bee was given a number. This numbering was used as random effect of the model. To test the distribution of choices between the four stimuli of each stage, a multiple comparison of means was done with the Tukey all-pair comparisons.

For the comparison of two-data samples, the Student’s *t*-test or the Mann-Whitney-*U* test were used.

## Results

**Fig 2:**
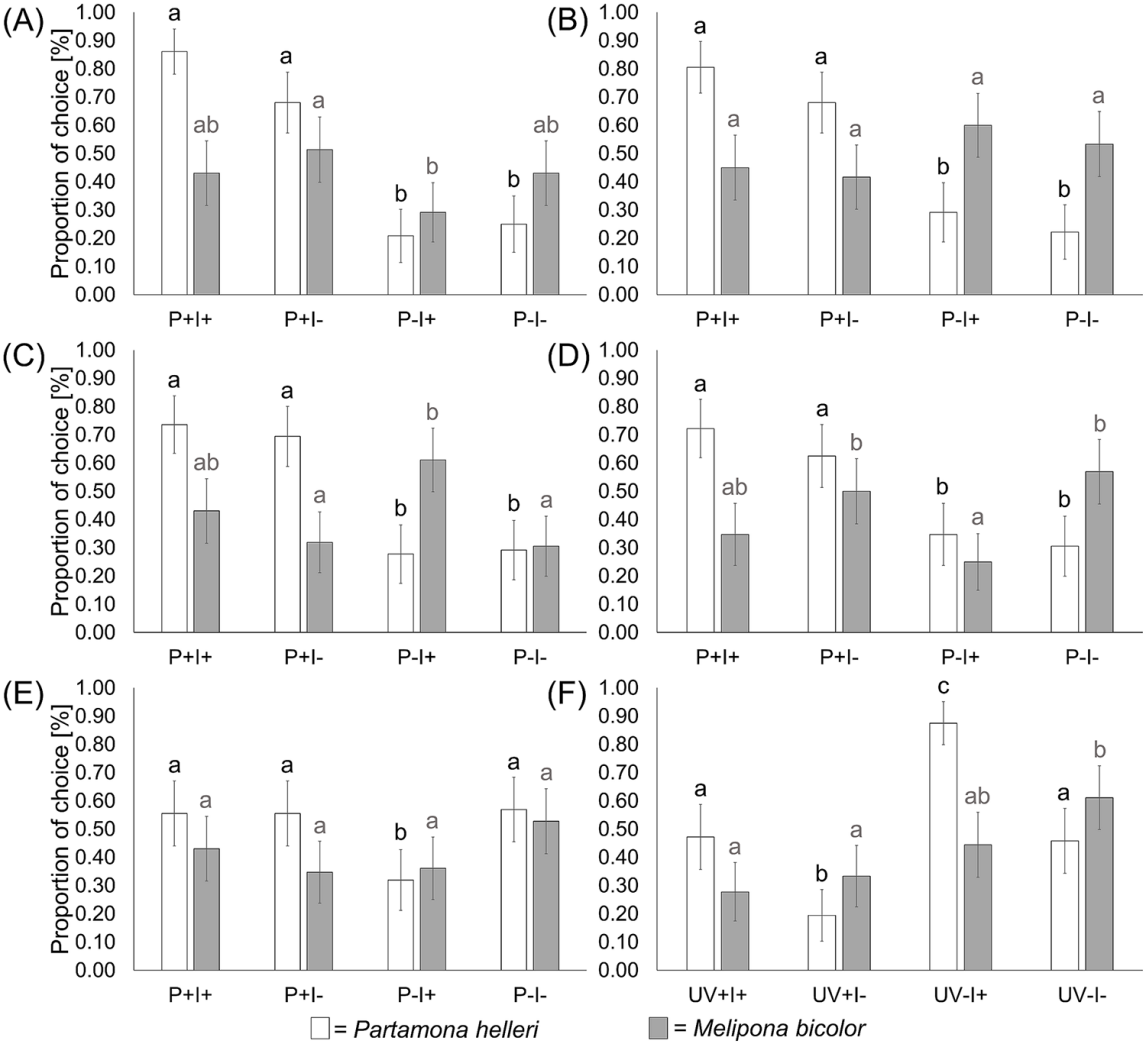
Colour choices within the colour categories. Six categories of colours were tested (A) = UV-blue, (B) = blue, (C) = UV-yellow, (D) = yellow, (E) = red and (F) = white. Each colour category consists of four stimuli with different levels of spectral purity (P+ = high spectral purity; P- = low spectral purity) and colour intensity (I+ = high colour intensity; I- = low colour intensity). Only in the white colour category spectral purity is replaced by UV properties of colours (UV+ = UV-reflecting; UV- = UV-absorbing). The total choices of *Partamona helleri* (black columns; n = 24) and *Melipona bicolor* (grey columns, n = 20) were compared by using a GLMM with Tukey’s all pair comparisons as post-hoc test. Different letters above the columns show statistical significances, where the same letters represent no significant results and different letters represent significant results. Error bars indicate binomial confidence intervals.

**Fig 3:**
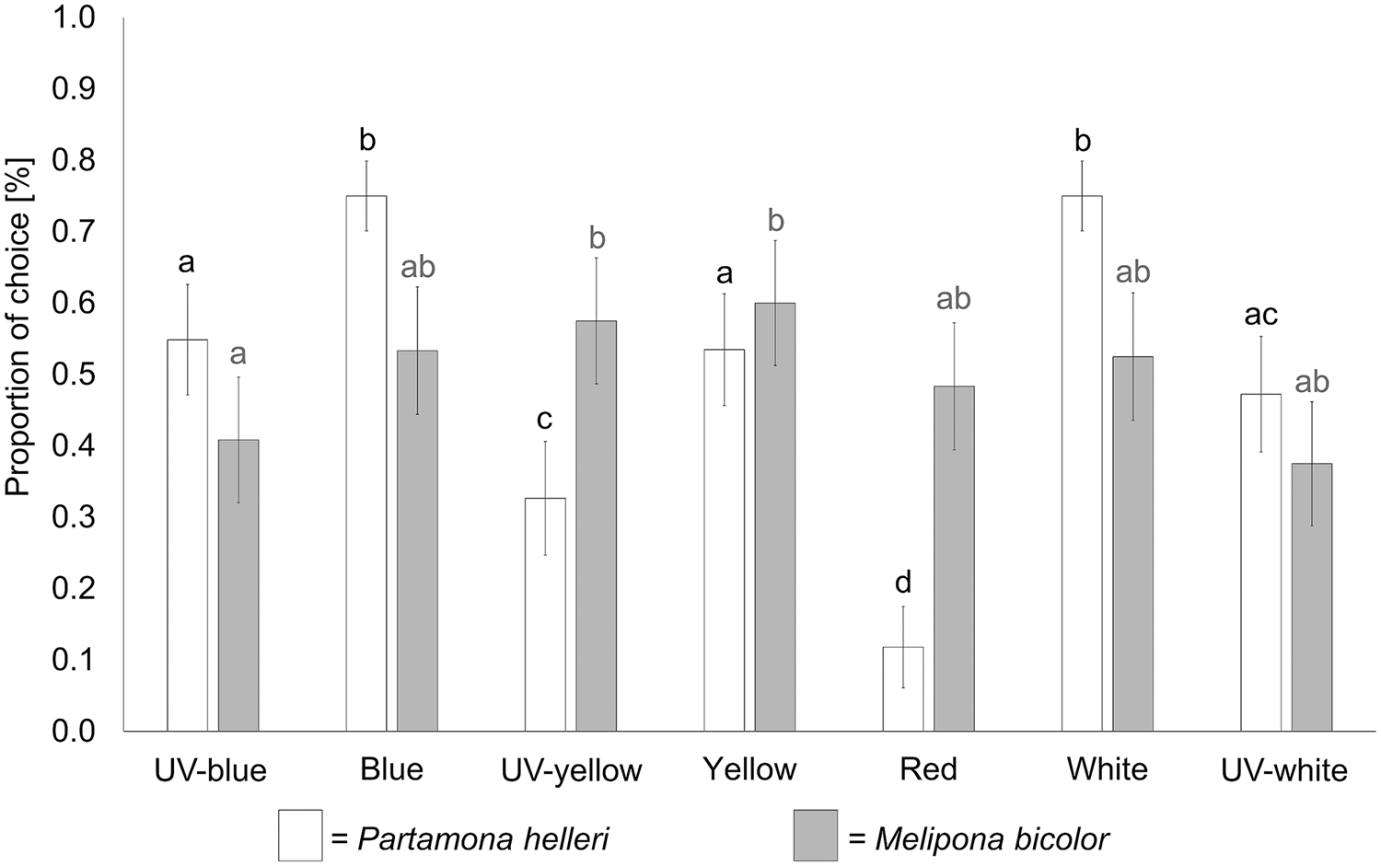
Colour choices according to differences in dominant wavelength. Seven dominant wavelengths (UV-blue, blue, UV-yellow, yellow, red, white and UV-white) were tested in dual choice experiments (each individual choose 21 times). The total number of choices of *Partamona helleri* (black columns; n = 24) and *Melipona bicolor* (grey columns, n = 20) were compared by using a GLMM with Tukey’s all pair comparisons as post-hoc test. Different letters above the columns show statistical significances, where the same letters represent no significant results and different letters represent significant results. Error bars indicate binomial confidence intervals.

Statistical analysis of colour choice behaviour for *Melipona bicolor* and *Partamona helleri* reveals that both species chose colours differently. Workers of *M*. *bicolor* do not show clear preferences within any of the tested colour categories (Fig 2). Only for the white colour category the UV-absorbing stimulus with reduced intensity was preferred by *M*. *bicolor* as well as the more intense stimuli in UV-yellow category. Concerning dominant wavelength, workers of *M*. *bicolor* showed no distinct preferences (Fig 3).

Workers of *P*. *helleri* showed strong differences in their choice behaviour compared to workers of *M*. *bicolor* (Fig 2). The stimuli with high spectral purity were generally preferred over less spectrally pure stimuli in the UV-blue, blue, UV-yellow and yellow colour category. In the red colour category, no preference for any of the stimuli could be observed. Furthermore, workers of *P*. *helleri* preferred UV-absorbing white colours over UV-reflecting white ones and also preferred UV-absorbing white and blue stimuli (both stimuli were chosen in 108 of 144 executed dual choice tests, n = 24, 6 dual choices per colour) over the other dominant wavelengths (Fig 3). Red was chosen least compared to the other dominant wavelengths (only chosen in 17 of 144 executed dual choice tests, n = 24, 6 dual choices per colour).

**Fig 4:**
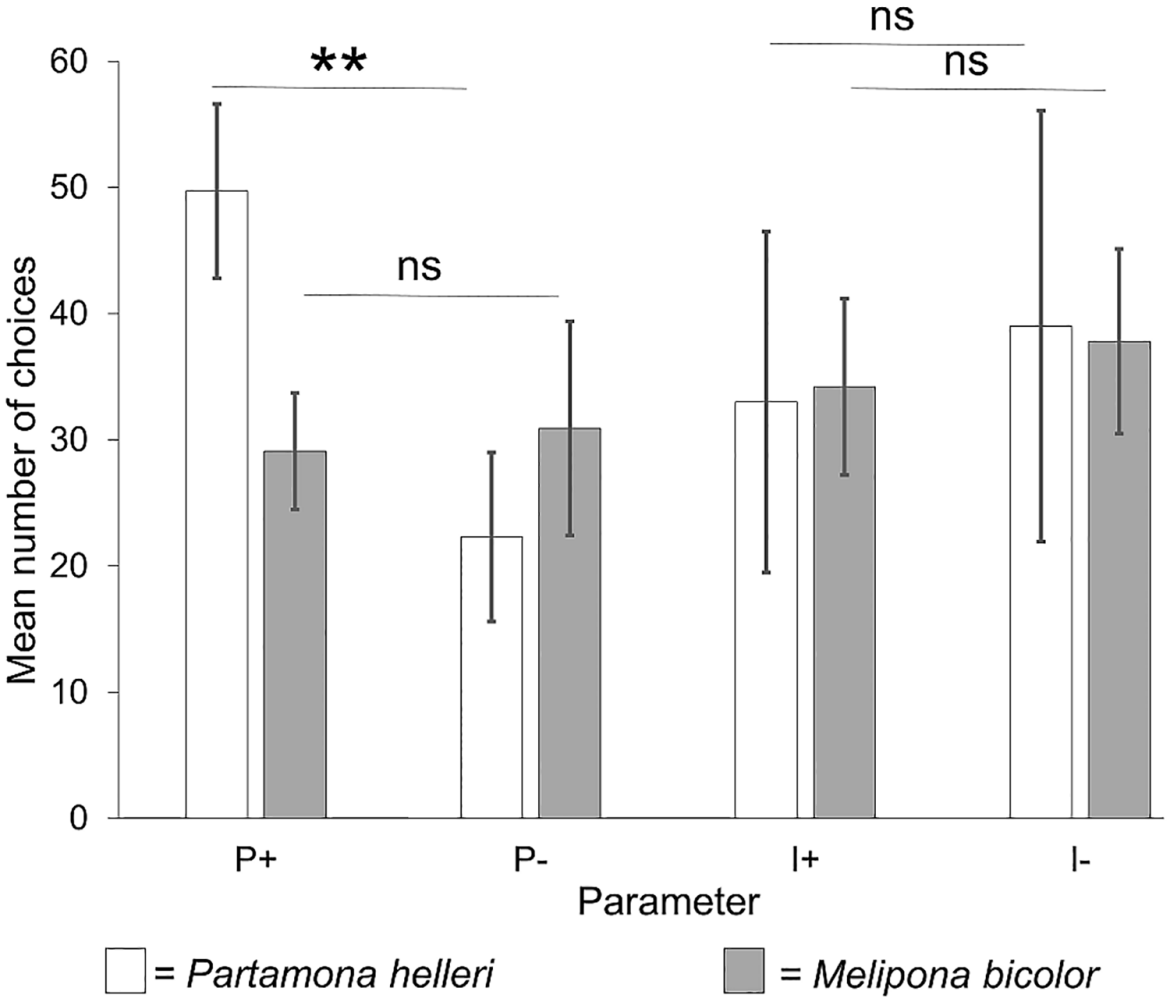
Mean number of choices according to spectral purity or intensity independent of dominant wavelength. The level of high spectral purity (P+) differs significantly when compared to the low spectral purity level (P-) for *Partamona helleri* (n = 24). For stimuli that differ in colour intensity the choices reveal no significant preferences for neither *P*. *helleri* nor *Melipona bicolor* (n = 20).

To analyse the effect of spectral purity and colour intensity on the colour choice behaviour of both stingless bee species, the total choices of spectrally purer colours (P+) were compared to the total choices for less spectrally pure colours (P-) and the total number of choices for more intense colours (I+) against the total number of less intense colours (I-) without considering dominant wavelength (Fig 4). None of the tested parameters elicit a distinct colour choice in *M*. *bicolor* (spectral purity: *t* = -0.6589, df = 9, p = 0.5264; intensity: *t* = -0.8655, df = 11, p = 0.4053, Student’s *t*-test) while *P*. *helleri* chose spectrally purer colours significantly more often than less pure colours (W = 98, p = 0.0003, Wilcox test) but do not pick colours based on intensity (W = 93, p = 0.236, Wilcox-test).

## Discussion

In the current study, we observed a preference for spectrally purer colours for workers of *Partamona helleri*, while *Melipona bicolor* generalized colours independent of dominant wavelength, intensity and spectral purity. In a previous study, *Melipona mondury* and *Melipona quadrifasciata* were tested concerning their colour preferences and similar results were obtained [21]. Both *Melipona* species chose colours independently of intensity and spectral purity and only minor preferences for UV-blue (*M*. *mondury*) or yellow (*M*. *quadrifasciata*) could be obtained.

Floral colour is one of the strongest advertisements by flowering plants and constitutes a long distance effect of flowers on flower visitors. In order to locate flowers bees need specific mechanisms to detect and recognize colours to collect food rewards most effectively. In honeybees and bumblebees dominant wavelength (≙ colour hue) and spectral purity (≙ saturation) were identified as main colour parameters influencing honeybees’ and bumblebees’ choice as well as colour contrast to the background and green contrast [17–19,28–30]. So far, little is known about colour preference in stingless bees. Dyer et al. [20] found a preference for UV-absorbing white colours in *Tetragonula carbonaria*, but more specific data concerning a preference for specific colour parameters like spectral purity or intensity (≙ brightness) could not be found yet [21].

One possible reason for the differences in the spontaneous colour choice between *M*. *bicolor* and *P*. *helleri* could be the recruitment behaviour of workers in these two species. Stingless bees are known to use chemical communication and chemical marking to exploit food sources [47]. Especially for high-density foragers, like *Melipona* and *Partamona*, chemical communication is important to recruit nest mates to the direction where rewarding food sources are located and the position of a food source itself. Naïve workers can either act as scout bees or as recruits that are informed by other scout bees [15]. In the experiments, the stimuli presented in a test were cleaned after each use so that chemical communication via scent-marked stimuli should not influence the experimental outcome. Each worker of *M*. *bicolor* had to be trained individually to the test arena because the tested workers did not recruit other workers, while *P*. *helleri* workers were frequently recruited by tested bees. The missing recruitment of *M*. *bicolor* could be explained by the small distance between hive and test arena (approximately 1.5 m). Species of the genus *Melipona* mark their food sources directly but do not place chemical markings along the way to a food source and a short way like 1.5 m could be insufficient to guide other workers from the nest entrance to the food source [48].

Another reason for the dissimilarity between the colour choices of the two bee species could be the different size of the colonies. *P*. *helleri* hives harbour up to 10000 individuals (personal communication Sergio Dias Hilário, USP), while *M*. *bicolor* hives only harbour up to 1000 individuals [49,50]. This difference in number could raise the pressure on *M*. *bicolor* being more generalistic than *P*. *helleri*.

The observed preference for spectrally purer colours in *P*. *helleri* accords to results observed in flower-experienced honeybees and bumblebees where workers spontaneously preferred spectrally purer colours of the same dominant wavelength independent of their conditioning [29, 30]. A field study in Greece showing a correlation between the amount of produced nectar and the spectral purity values of floral colours suggests that a preference for spectrally purer colours by bees could be advantageous to find higher rewarding flowers [51]. The choices concerning dominant wavelength of *P*. *helleri* assort to known preferences in honeybees, bumblebees and Australian stingless bees which showed preferences for bluish colours [20,28,32].

The calculated values for intensity (bee-subjective vision) and luminance (physical values) are in accordance with each other, while the values for spectral purity (bee-subjective vision) and saturation (physical values) only resemble each other for the red stimuli (see S1 Table). While the obtained results *for P*. *helleri* support the bee-subjective values calculated with the hexagon model by Chittka [37] the results obtained for *M*. *bicolor* can be explained with neither physical nor bee-subjective calculations. Based on the choices of *P*. *helleri* that can only be explained by the colour hexagon this model appears to be a solid method for the calculation of spectral purity.

Many studies analysed colour choice in hummingbirds and found that experienced hummingbirds showed preferences for red colours but naïve hummingbirds show no spontaneous preferences for specific colours and instead rather decide for location or quality of a food source [52–58]. Furthermore, a study by Lunau et al. [59] observed the absence of colour preferences in hummingbirds for UV-absorbing red and UV-reflecting white flowers, though these are typical floral colours of hummingbird pollinated flowers [60–62]. The results of that study suggest that hummingbirds engage a private niche that is created by the inability of other pollinators (in this case orchid bees) to detect these floral colours. This “bee avoidance” hypothesis has been confirmed in the field by Bergamo et al. [63]. So far, all experimental testing of colour preferences in the genus *Melipona* (three species *M*. *bicolor*, *M*. *mondury* and *M*. *quadrifasciata*) could only show slight preferences for specific colours with no pervading pattern [21]. In this view, *Melipona* developed different mechanisms to locate food sources other than colour perception and is thus less excluded by flower colours of low spectral purity that specifically allure hummingbirds, i.e. UV-reflecting white and UV-absorbing red.

In total, these results show that a generalization of colour preferences in bees is misleading since *M*. *bicolor* and *P*. *helleri* show strong differences in their colour choices. *M*. *bicolor* shows no colour choice behaviour, while *P*. *helleri* shows a similar colour choice behaviour in comparison to honeybees and bumblebees. Flower detection in *Melipona* seems to be less dependent on colour vision than on other criteria like chemical marking, odour or location of food sources.

## Supporting information

S1 Fig Experimental setup. (TIF)

S1 Table Mixture ratios of colour pigments and calculated colour parameters of compacted stimuli. (Black* = see stimulus Black; Grey* = see mixture Grey; Yellow-grey* = see mixture Yellow-grey; P+ = high spectral purity; P- = low spectral purity; I+ = high intensity; I- = low intensity; UV- = UV-absorbing; UV+ = UV-reflecting).
(TIF)

S2 Table Semi-randomized order of dual choice tests. (UVB = UV-blue; B = blue; UVY = UV-yellow; Y = yellow; W = white; R = red; P+ = high spectral purity; P- = low spectral purity; I+ = high intensity; I- = low intensity; UV- = UV-absorbing; UV+ = UV-reflecting).
(TIF)

S1 Data Raw data. (XLSX)
