# Supplementary figures and images for: Spectral purity, intensity and dominant wavelength: Disparate colour preferences of two Brazilian stingless bee species
Source: PLoS One. 2018 Sep 28;13(9):e0204663. doi: 10.1371/journal.pone.0204663 (PMC6162086; doi:10.1371/journal.pone.0204663)

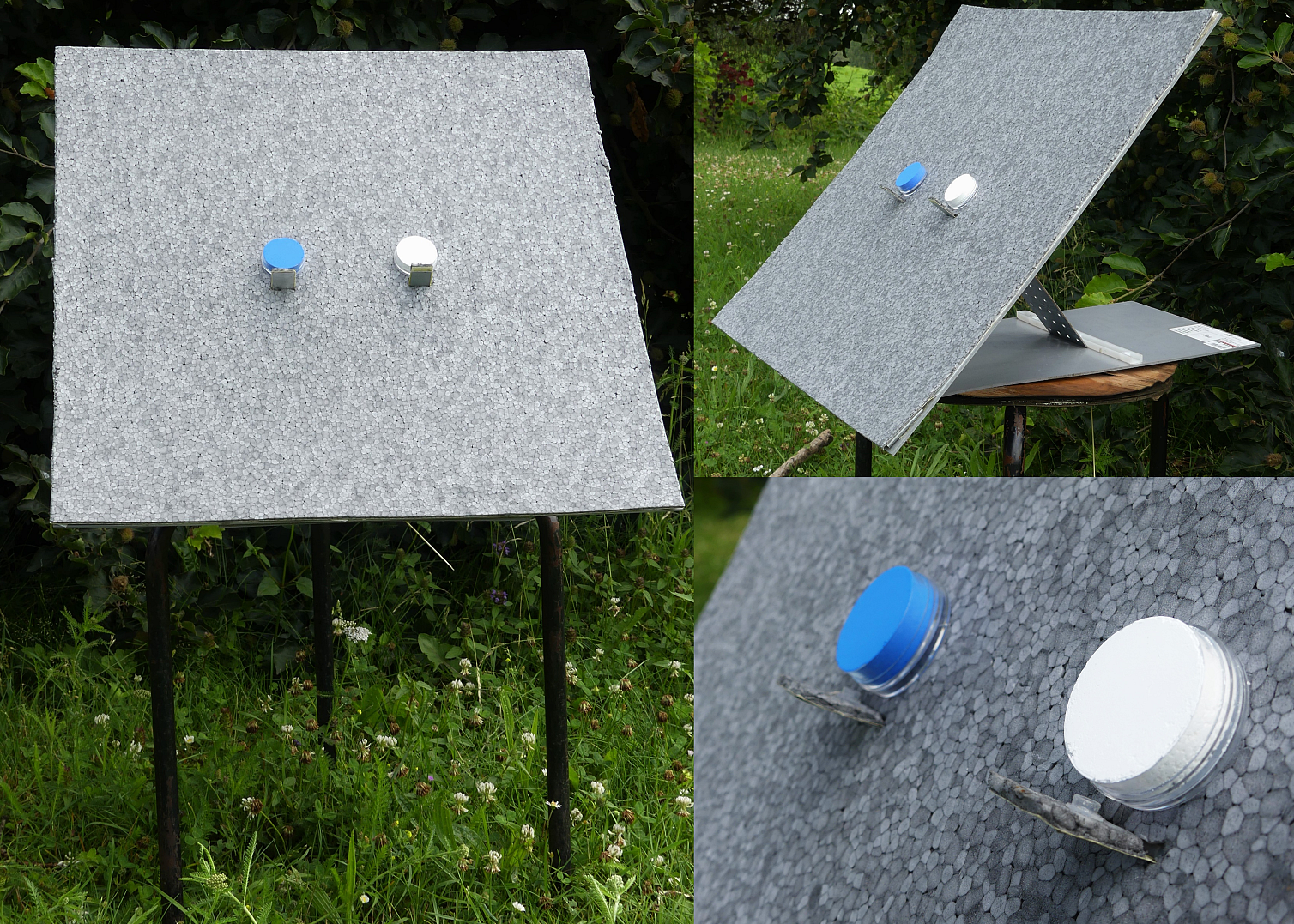

Supplement: S1 Fig — (TIF) [file pone.0204663.s001.tif]

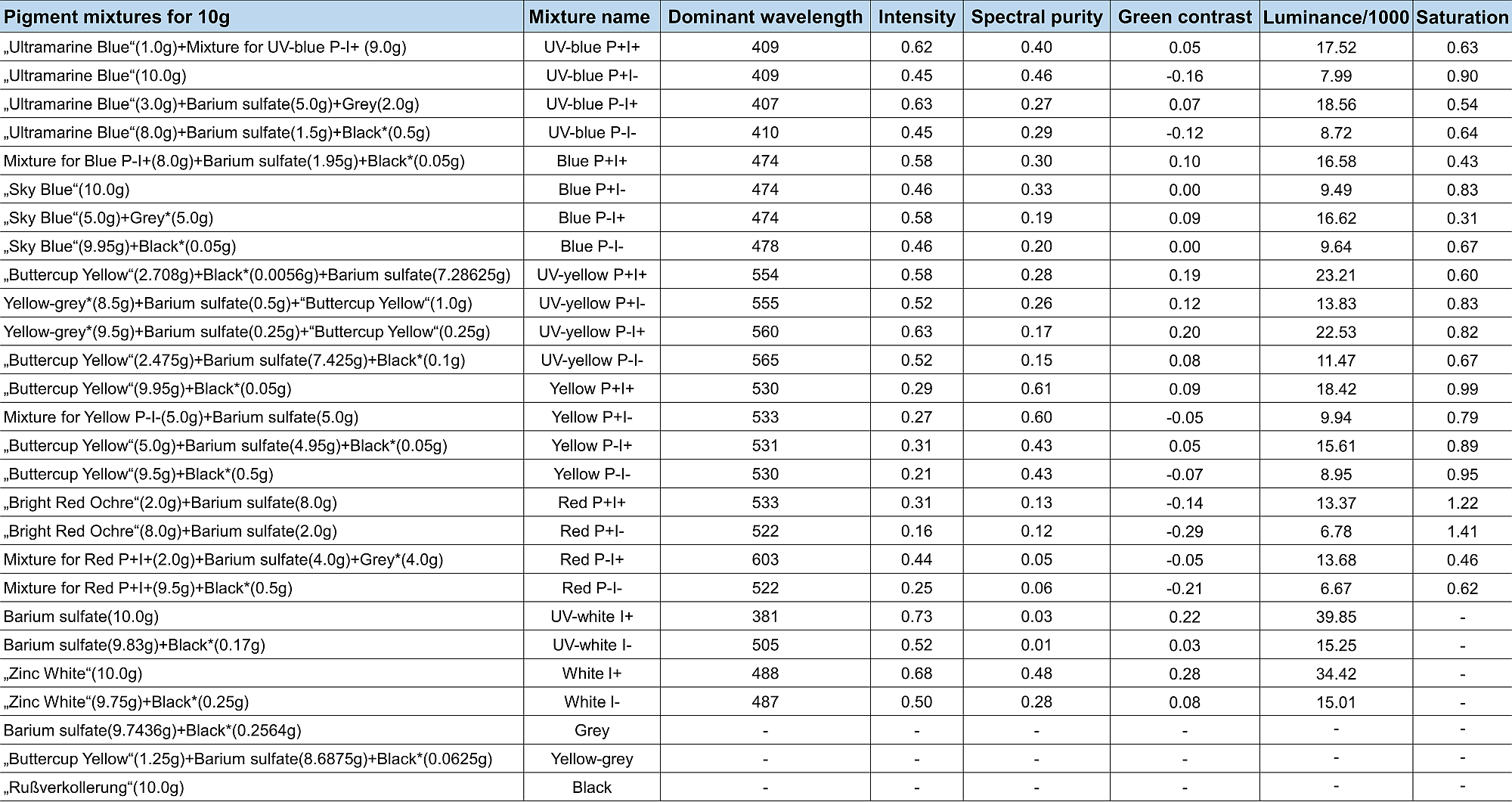

Supplement: S1 Table — (Black* = see stimulus Black; Grey* = see mixture Grey; Yellow-grey* = see mixture Yellow-grey; P+ = high spectral purity; P- = low spectral purity; I+ = high intensity; I- = low intensity; UV- = UV-absorbing; UV+ = UV-reflecting). (TIF) [file pone.0204663.s002.tif]

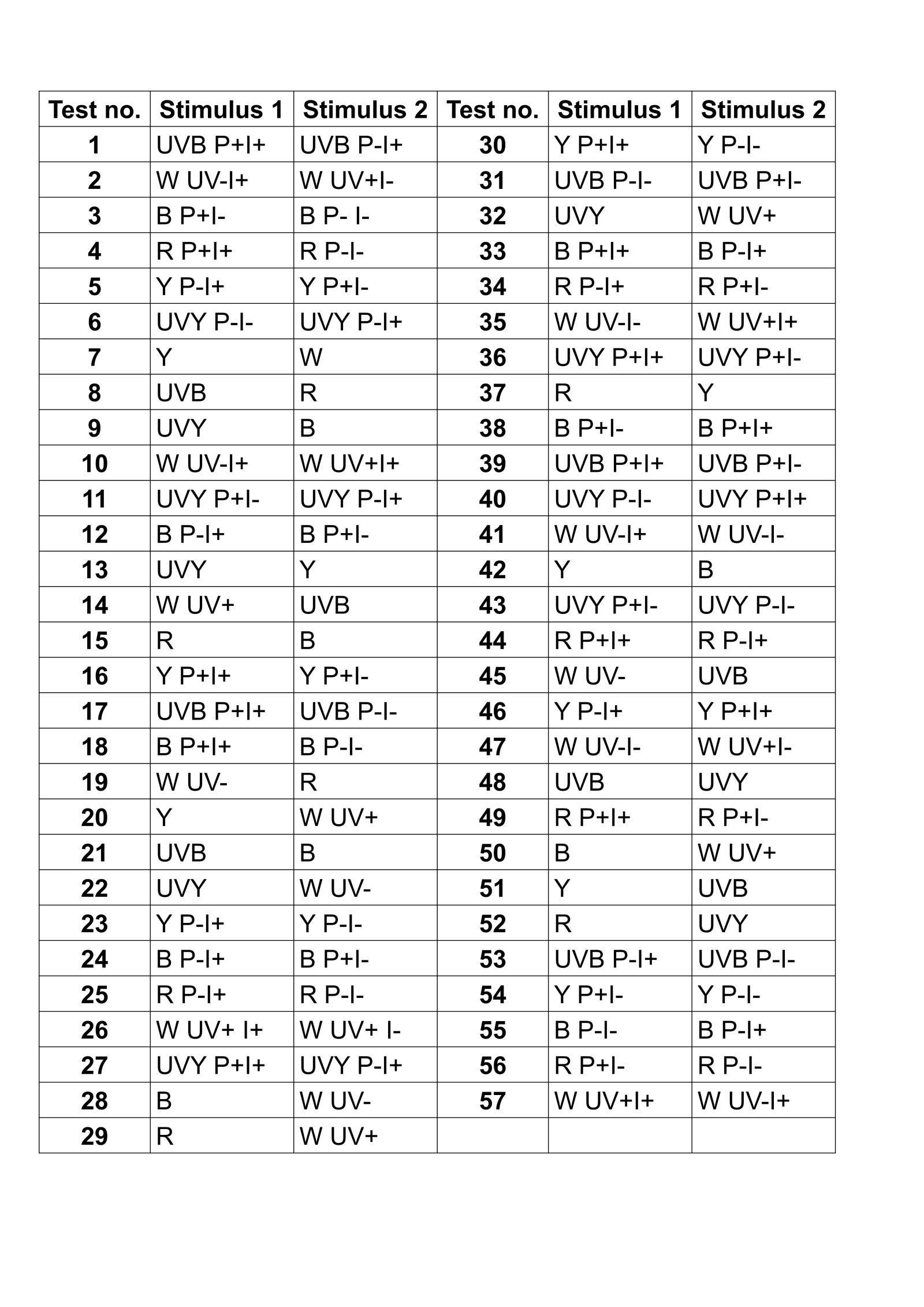

Supplement: S2 Table — (UVB = UV-blue; B = blue; UVY = UV-yellow; Y = yellow; W = white; R = red; P+ = high spectral purity; P- = low spectral purity; I+ = high intensity; I- = low intensity; UV- = UV-absorbing; UV+ = UV-reflecting). (TIF) [file pone.0204663.s003.tif]
